# Noninvasive local correction of loop ileostomy prolapse using a stapling device for a patient in the terminal phase of malignancy

**DOI:** 10.1093/jscr/rjz050

**Published:** 2019-03-05

**Authors:** Yutaka Kojima, Kazuhiro Sakamoto, Masaya Kawai, Kosuke Mizukoshi

**Affiliations:** Department of Coloproctological Surgery, Faculty of Medicine, Juntendo University, Tokyo, Japan

## Abstract

A 64-year-old woman who underwent surgery for ovarian cancer, but were unable to be resected and were directly infiltrated at the terminal ileum, and the ileostomy in the oral side of the terminal ileum was performed as a palliative operation. Prolapse of the ileostomy appeared approximately 17 months after the operation, and after 19 months, the blood circulation disorder of the intestinal tract and the reduction of prolapse became difficult. Since the patient’s systemic condition was poor, anesthesia was not enforced, and an ileostomy reconstruction was performed using a stapling device while performing mild sedation. During the operation, the patient was unconscious, and the operation time was 29 minutes, and the general condition was not abnormal. In this paper, we report noninvasive local correction of loop ileostomy prolapse using a stapling device for a patient in the terminal phase of malignancy.

## INTRODUCTION

Stomal prolapse is one of the late complications of ileostomy or colostomy. Prolapse is a common complication, with loop stoma occurring in 2–22% of cases [1], and it can compromise the patients’ quality of life. We report the noninvasive local correction of loop ileostomy prolapse using a stapling device for a patient in the terminal phase of malignancy.

## CASE REPORT

A 64-year old female with unresectable ovarian carcinoma underwent resection of the seeding nodule of ovarian carcinoma and created a loop ileostomy in the right lower part of the abdomen due to direct invasion of the carcinoma to the terminal ileum. During the formation of the loop ileostomy, the ileum penetrated the rectus abdominis muscle; the ileum was fixed to the anterior sheath of the rectus abdominis muscle with 8–10 stitches. Furthermore, the ileum and skin were also fixed with 12 stitches. There was no fixation between the peritoneum and the mesentery of the ileum. The patient subsequently underwent chemotherapy. Prolapse of the ileostomy appeared approximately 17 months after the operation, and it continued to progressively worsen. Repositioning of the prolapse was especially difficult, as bleeding occurred from the mucosa of the prolapsed intestine, and there were edematous and ischemic changes of tip of prolapsed intestine. Thus, we decided to perform the operation. The patient’s Performance Status was three and the general state was gradually getting worse. Pethidine hydrochloride (17.5 mg) was administered intravenously to obtain pain relief just before the operation. No heavy sedative was prescribed for the patient and, while conscious, the patient remained lucid throughout the operation.

**Figure 1:**
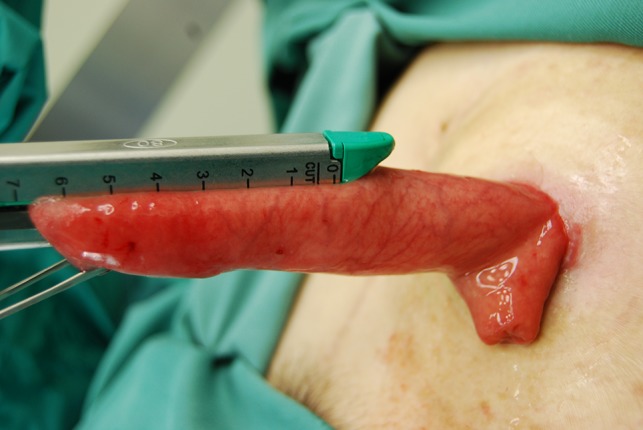
The prolapsed intestine was cut along the axis intersecting perpendicular to the mesentery by GIATM 60.

The prolapsed intestinal tract with the Alice forceps was cut in accordance with the axis that intersected perpendicular to the mesentery by GIA^TM^ 60-4.8 (COVIDIEN, Dublin, IRL) (Fig 1a and b). It was separated so that the height of the intestinal tract that remained might be set 4–5 cm from the skin. Next, the isolated intestinal tract was separated using the same device in the direction of the minor axis (Fig. 1c). The interrupted suture was carried out to reinforce the part at which the stapler overlapped using absorbable sutures, and the operation was completed (Fig. 1d). The postoperative progress was good, and the chemotherapy was continued. The patient’s general state got worse gradually, and her treatment became a plan of best supportive care. Nineteen months after the first time operation, the general state got even worse and she died. After revision of the prolapse, there were no further troubles with the ileostomy.

## DISCUSSION

**Figure 2:**
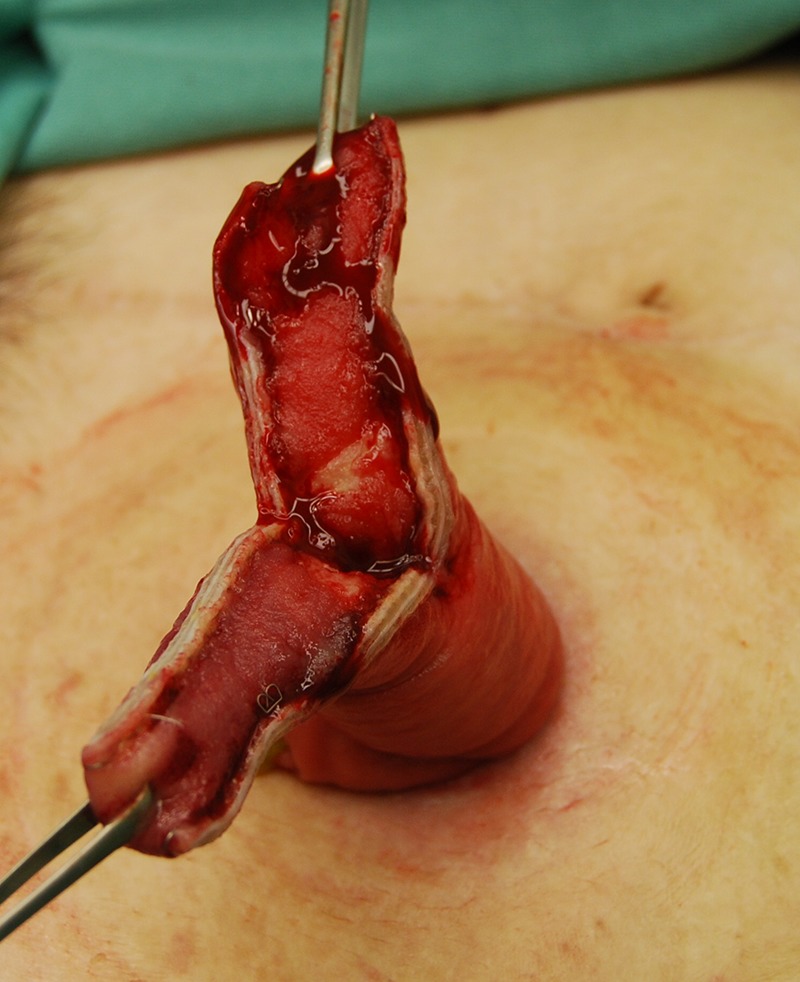
The prolapsed intestine was cut in two places in the major axis direction.

**Figure 3:**
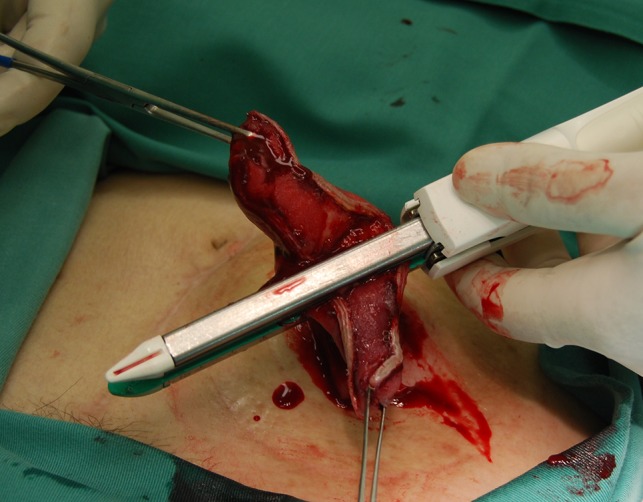
The isolated intestinal tract was separated using the same device as the direction of the minor axis.

**Figure 4:**
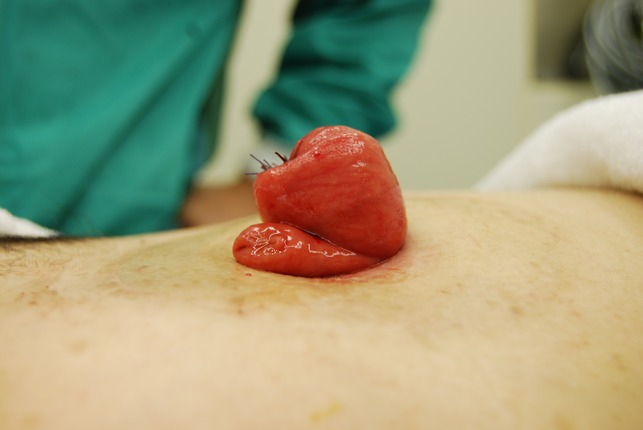
The operation was completed.

Stoma formation is commonly performed in operations for malignant disease, inflammatory bowel disease, etc. Stoma formation can be either temporary or permanent, ileostomy or colostomy, and these can be end or loop stomas. While it is a simple procedure, complications are common. Stomal prolapse is one of the late stoma complications. The reported incidences of prolapse for ileostomy and colostomy are 8–75% and 5.4–18%, respectively [2, 3]. Stomal prolapse has been reported to occur in loop stomas more often than end stomas and commonly involves the distal limb [4, 5]. The distal intestinal tract, which does not contribute to the pathogenesis, undergoes disuse withering, and we believe that it is easy for prolapse to occur by becoming smaller than the initial size at the time of stoma formation. Stomal prolapse can cause distress for the patient but it is usually of no clinical or functional significance. Rarely, prolapse can cause obstruction, increase the risk of infarction, skin irritation and difficulties with appliance management [1, 6]. Various etiological factors have been postulated for stomal prolapse, including obesity, a large opening in the abdominal wall at the time of stoma formation, conditions causing increased intra-abdominal pressure and a redundant loop of the bowel proximal to the stoma [6]. It does not seem to matter whether or not the stoma is primarily constructed with fixation of the mesentery to the peritoneum of the anterior abdominal wall, because stoma prolapse is most commonly of the sliding type [7]. In our case, there was no obvious cause of the stoma prolapse. Several techniques have been described to revise stoma when prolapse occurs, which include simple refashioning, re-sitting and laparotomy, button-plexy fixation, etc. [1, 8] After stapler devices appeared, revision using stapler have been reported in a small series for prolapse of loop colostomy, loop ileostomy and rectum [1, 9, 10]. When the small intestine was cut by GIA^TM^ 60-4.8, since the thickness of the intestinal wall or mesentery was thicker than the large intestine, a 4.8 mm high staple quantity was used in our case. Furthermore, the small intestine had good blood flow, the portion with which the staple overlapped was sutured to prevent hemorrhage. As in our case, patients with unresectable cancer often require stoma formation and their general state is poor in many cases. This procedure could be safely enforced with brief periods of minor intravenous sedation, and was considered to be useful for patients whose general state was poor. This procedure is easy to perform, does not require general anesthesia, and can be performed at the bedside. However, it is important to note that stapling across a prolapsed stoma is a relatively blind procedure, and extra caution must be used to avoid entrapment of the bowel loops from an unrecognized parastomal hernia [10]. Thus, before the revision of stomal prolapse it is necessary to check for the presence of a parastomal hernia by computed tomography scan. Although the use of a stapler is more costly, this procedure may be an option for the management of stomal prolapse and can be used to avoid laparotomy and allow local repair with reconstruction of the loop ileostomy for patients with poor general outcome and requiring palliative care (Figs 2–4).

## References

[1] Skaerlund ML, Jacobsen L, Tottrup A Ileostomy revision using noncutting linear stapler. Colorectal Dis 2008;10:833–6. 18325045 10.1111/j.1463-1318.2007.01458.x

[2] Robertson I, Eung E, Hughes D, Spires M, Donnelly L, Mackenzie I, et al Prospective analysis of stoma related complications. Colorectal Dis 2005;7:279–85. 15859968 10.1111/j.1463-1318.2005.00785.x

[3] Londono-Schimmer EE, Leong APK, Philips RKS Life table analysis of stomal complications following colostomy. Dis Colon Rectum 1994;37:916–20. 8076492 10.1007/BF02052598

[4] Chandler JG, Evans BP Colostomy prolapsed. Surgery 1978;84:577–82. 715672

[5] Fucini C A simple device for prolapsing loop colostomies. Dis Colon Rectum 1989;32:534–5. 2791792 10.1007/BF02554514

[6] McErlain D, Kane M, McMgrogan M, Haughey S Prolapsed stoma. Nurs Stand 2004;18:41–2. 14768232

[7] Leong AP, Londono-Schimmer EE, Phillips RK Life-table analysis of stomal complications following ileostomy. Br J Surg 1994;81:727–9. 8044564 10.1002/bjs.1800810536

[8] Canil K, Fitzgerald P, Lau G, Cameron G, Walton M Button-pexy fixation for repair of ileostomy and colostomy prolapse. J Pediatr Surg 1995;30:1148–9. 7472969 10.1016/0022-3468(95)90008-x

[9] Maeda K, Maruta M, Utsumi T, Sato H, Aoyama H, Katsuno H, et al Local correction of a transverse loop colostomy prplapse by means of a stapler device. Tech Coloproctol 2004;8:46–6. 10.1007/s10151-004-0051-y 15057590

[10] Roland S, Lukas M, Franc HH Perineal stapled prolapse resection: a new procedure for external rectal prolapse. Dis Colon Rectum 2008;51:1727–30. 18626711 10.1007/s10350-008-9423-0

